# Identification of key apoptosis-related genes and immune infiltration in the pathogenesis of psoriasis

**DOI:** 10.1186/s41065-022-00233-0

**Published:** 2022-06-22

**Authors:** Ailing Zou, Qingtao Kong, Hong Sang

**Affiliations:** 1 The First School of Clinical Medicine, Southern Medical University, Guangzhou, 510515 China; 2 Department of Dermatology, Huangshi Central Hospital, Affiliated Hospital of Hubei Polytechnic University, Edong Health Care Group, Huangshi, 435000 China; 3 Department of Dermatology, Jinling Hospital, Nanjing, 210002 China

**Keywords:** Psoriasis, Pathogenesis, DEARGs, Immune infiltration

## Abstract

**Background:**

Psoriasis is a condition in which skin cells build up and form itchy scales and dry patches. It is also considered a common lifelong disease with an unclear pathogenesis. Furthermore, an effective cure for psoriasis is still unavailable. Reductive apoptosis of keratinocytes and immune infiltration are common in psoriasis. This study aimed to explore underlying functions of key apoptosis-related genes and the characteristics of immune infiltration in psoriasis. We used GSE13355 and GSE30999 to screen differentially expressed apoptosis related genes (DEARGs) in our study. Gene Ontology (GO), Kyoto Encyclopedia of Genes and Genomes (KEGG) pathway, and gene set enrichment analysis (GSEA) were performed using clusterProfiler package. Protein–protein interaction (PPI) network was constructed to acquire key DEARGs. Transcription factor (TF)–target and miRNA–mRNA network analyses, drug sensitivity prediction, and immune infiltration were applied. Key DEARGs were validated using real-time quantitative PCR (RT-qPCR).

**Results:**

We identified 482 and 32 DEARGs from GSE13355 and GSE30999, respectively. GO analysis showed that DEARGs were commonly enriched in cell chemotaxis, receptor ligand activity, and signaling receptor activator activity. KEGG pathway analysis indicated that viral protein interaction with cytokine and cytokine receptor was maximally enriched pathway. The GSEA analysis of GSE13355 and GSE30999 demonstrated a high consistency degree of enriched pathways. Thirteen key DEARGs with upregulation were obtained in the PPI network. Eleven key DEARGs were confirmed using RT-qPCR. Additionally, 5 TFs and 553 miRNAs were acquired, and three novel drugs were predicted. Moreover, Dendritic.cells.activated exhibited high levels of immune infiltration while Mast.cells.resting showed low levels of immune infiltration in psoriasis groups.

**Conclusion:**

Results of this study may reveal some insights into the underlying molecular mechanism of psoriasis and provide novel targeted drugs.

**Supplementary Information:**

The online version contains supplementary material available at 10.1186/s41065-022-00233-0.

## Background

Psoriasis is a common lifelong dermatological disease with prevalence varied from 0.14% to 1.99% of the population worldwide [1, 2]. Approximately 125 million people worldwide suffer from psoriasis, and this number continues to show a gradual increasing trend [3–5]. Psoriasis is often associated with systemic illnesses, such as hypertension, diabetes, and coronary heart diseases [3, 6], and remarkably reduces the physical and mental health and quality of life while increasing the economic burden of patients [3]. Psoriasis is a polygenic disease caused by the interaction of multiple factors, such as genetics and environment [6, 7]. Although the exact pathogenesis of psoriasis is unclear, abnormal proliferation of keratinocytes and disruption of the immune system are central to its pathogenesis [8]. Therefore, exploring gene functions and underlying characteristics of immune infiltration in psoriasis is necessary.

Keratinocytes manifest abnormal proliferation and a marked inhibition of apoptosis [9]. The inhibition of apoptosis is related to imbalances in epidermal homeostasis and results in psoriatic hyperplasia [9, 10]. Thus, apoptosis-related genes (APRs) also play a vital role in the pathogenesis. Although mine differential genes have been extensively investigated with the progress of bioinformatics analysis, studies on the function of APRs are limited [11, 12]. For instance, Gao et al. revealed seven hub genes, namely, HERC6, ISG15, MX1, RSAD2, OAS2, OASL, and OAS3. Choudhary et al. reported the top 10 hub genes (CCNB1, CCNA2, CDK1, IL1B, CXCL8, MKI67, ESR1, UBE2C, STAT1, and STAT3) but they failed to distinguish between their properties.

Immune system imbalance plays an important role in the formation of psoriatic lesions [13]. Keratinocytes and innate immune cells, such as macrophages, plasmacytoids, and dendritic cells, are activated to secrete inflammatory factors (TNF-α, IFN-γ, etc.), which activate myeloid dendritic cells and then migrate to lymph nodes, under the stimulation of external factors [13, 14]. These dendritic cells can in turn activate T lymphocytes. Activated T cells secrete a variety of cytokines that interact with keratinocytes, neutrophils, and macrophages to induce a local persistent inflammatory response, which finally leads to the formation of psoriatic lesions [15].

In this study, we screened Differentially Expressed Apoptosis-Related Genes (DEARGs) associated with psoriasis and explored functions of DEARGs through a comprehensive bioinformatics analysis.

## Results

### Data selection and DEG screening

**Fig. 1:**
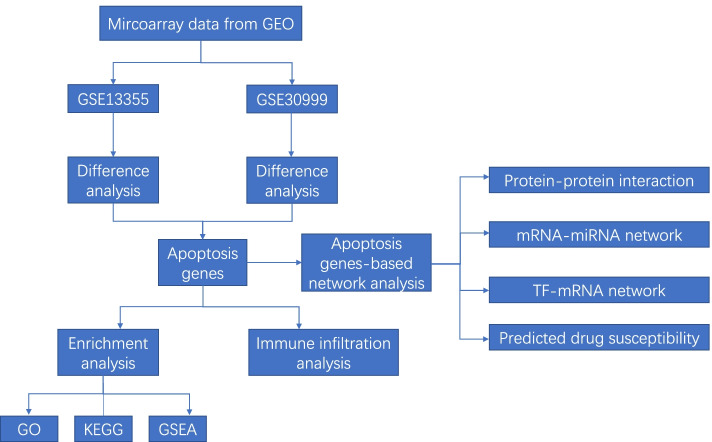
Flowchart of current study. GEO, Gene Expression Omnibus; GO, Gene Ontology; KEGG, Kyoto Encyclopedia of Genes and Genomes; GSEA, Gene Set Enrichment Analysis; TF, Transcription Factor

**Fig. 2:**
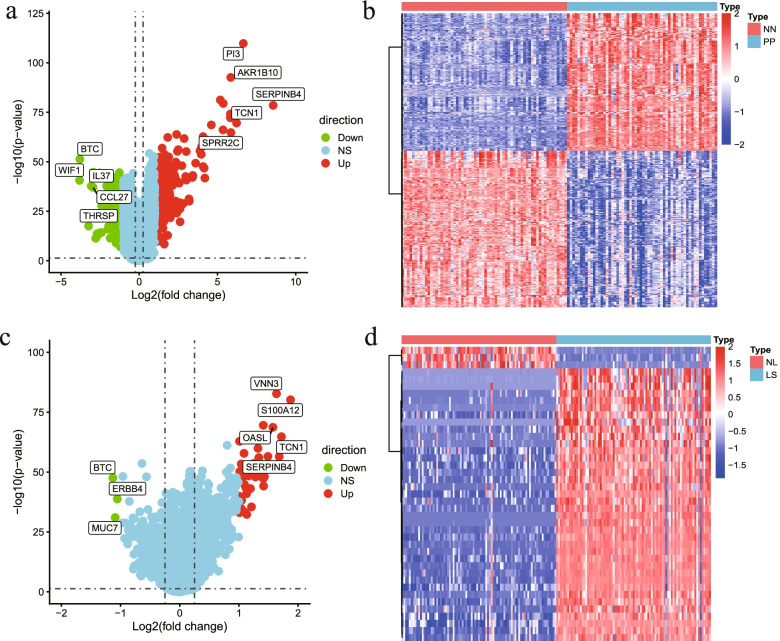
The differential analysis of GSE13355 and GSE30999. **a** Volcano plot of GSE13355 (Red, green and blue represent up-regulated, down-regulated and no differential genes, respectively). **b** Heatmap of GSE13355 (Blue indicates psoriasis group, and red indicates control group). **c** Volcano plot of GSE30999(Red, green and blue represent up-regulated, down-regulated and no differential genes, respectively). **d** Heatmap of GSE30999 (Blue indicates psoriasis group, and red indicates control group). NN, normal skin from controls; PP, involved skin from psoriatic patients; NL, non-lesional skin; LS, psoriasis lesions; NS, no significance

We first illustrated the flowchart of current study (Fig. 1), and then collated data according to the GEO data platform (Table 1). The comparison of psoriasis and control groups showed that the sample size is relatively balanced, thereby indicating the basis of statistical analysis. We obtained 297 up-regulated and 339 down-regulated genes using R software after normalizing the gene expression matrix of GSE13355, as shown in the volcano diagram in Fig. 2a. Additionally the top 10 genes with maximal significant differences were then annotated. The DEG heatmap of GSE13355 shows NN and PP represent control group and psoriasis group respectively (Fig. 2b). Similarly, 38 up-regulated and 3 down-regulated genes were obtained after normalizing GSE30999, as shown in the volcano diagram in Fig. 2c. The DEG heatmap of GSE30999 shows NL and LS represent control group and psoriasis group respectively (Fig. 2d).

**Table 1:** Data information summary

GEO accession	Platforms	Sample	
GSE13355	GPL570	NN	64
		PP	58
GSE30999	GPL570	NL	85
		LS	85

### Identification and functional analysis of DEARGs

**Fig. 3:**
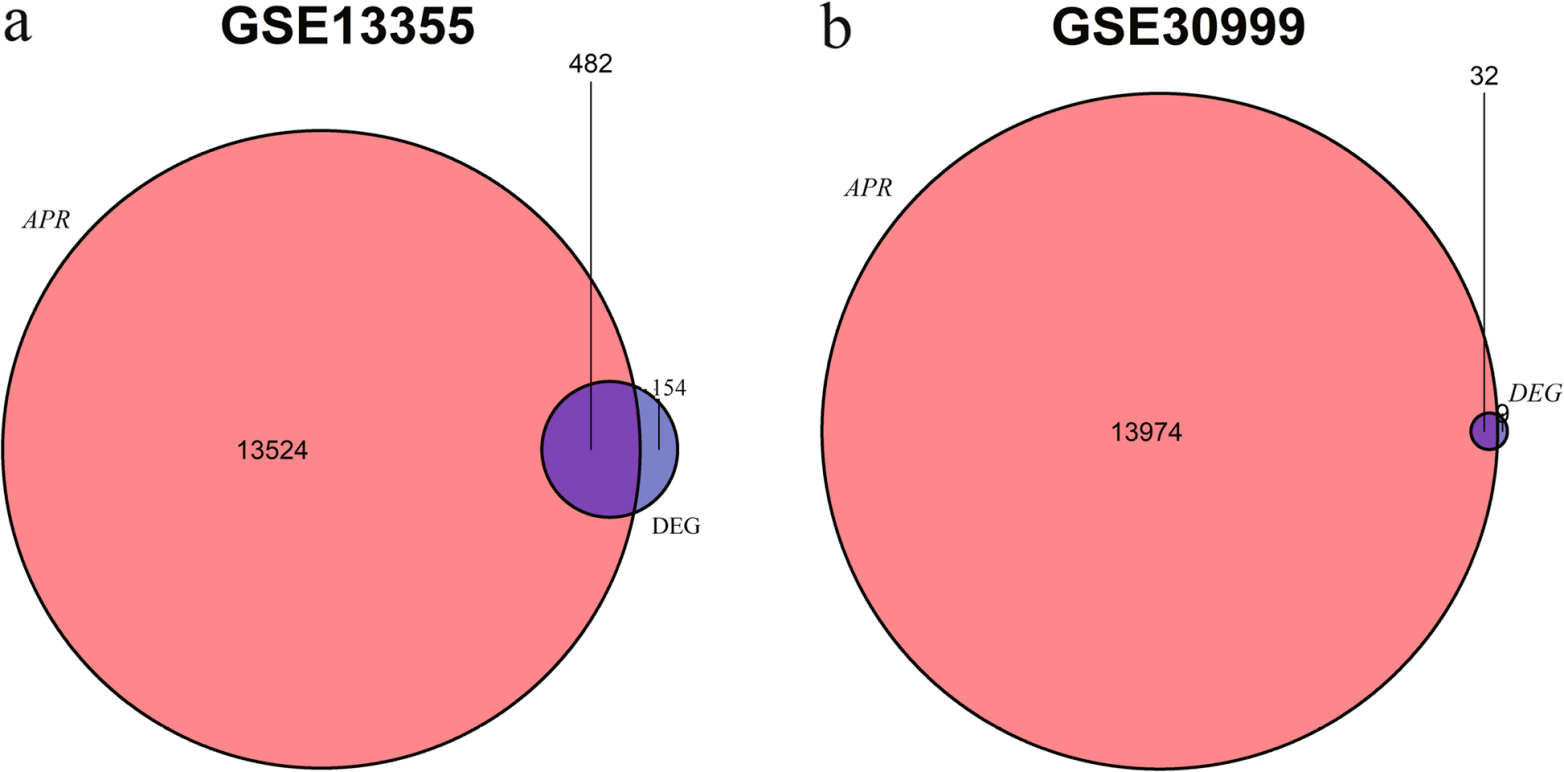
Screening differentially expressed apoptosis related genes (DEARGs) in two datasets via venn diagrams. **a** Intersection analysis of differentially expressed genes (DEGs) and apoptosis-related genes (ARGs) in GSE13355. **b** Intersection analysis of differentially expressed genes (DEGs) and apoptosis-related genes (ARGs) in GSE30999. APR, Apoptosis-Related Gene; DEG, Differentially Expressed Gene

**Fig. 4:**
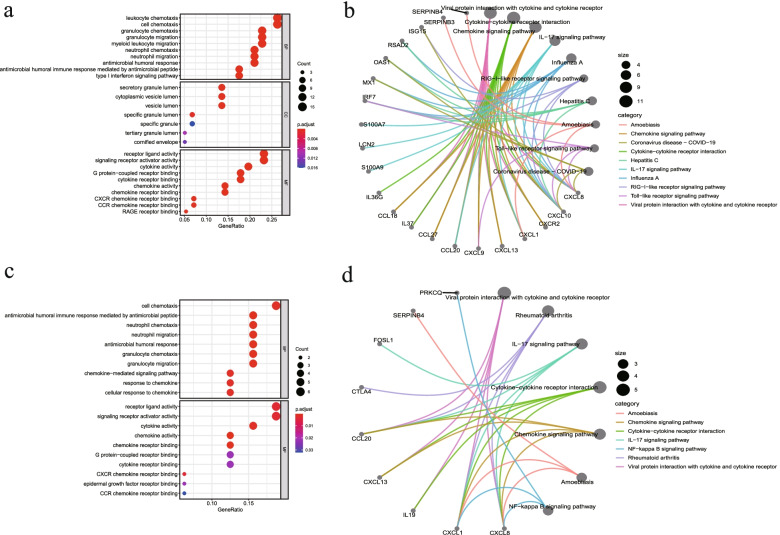
Functional analysis of DEARGs. **a** GO enrichment analysis of GSE13355. **b** KEGG pathway enrichment analysis of GSE13355. **c** GO enrichment analysis of GSE30999. **d** KEGG pathway enrichment analysis of GSE30999. (X horizontal axis represents the proportion of DEARGs enriched in GO team. The color of the dots indicates the adj.*p* value: the redder the color, the smaller the adj.*p* value; the bluer the color, the larger the adj.*p* value. The size of the dots implies the number of enriched genes). DEARGs, Differentially Expressed Apoptosis Related Genes; GO, Gene Ontology; KEGG, Kyoto Encyclopedia of Genes and Genomes

**Fig. 5:**
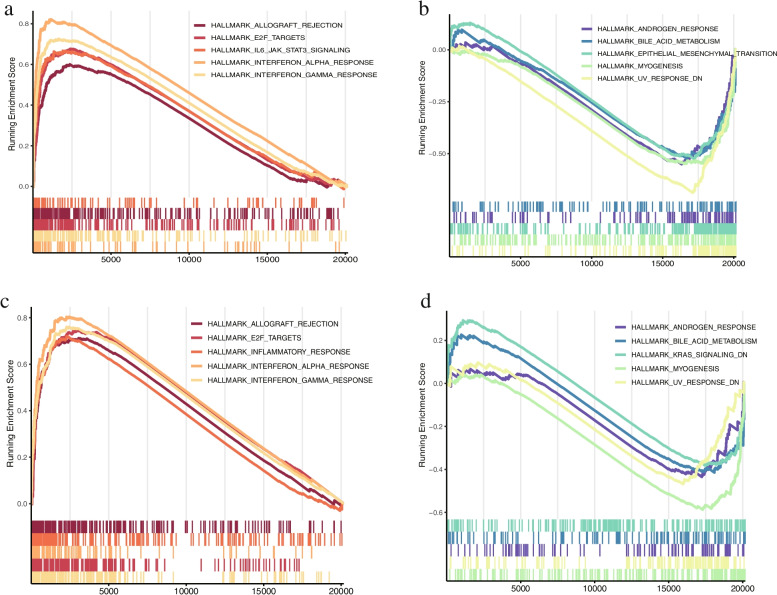
The GSEA results of GSE13355 and GSE30999. **a** The up-regulated enriched pathways in GSE13355. **b** The down-regulated enriched pathways in GSE13355. **c** The up-regulated enriched pathways in GSE30999. **d** The down-regulated enriched pathways in GSE30999. GSEA, Gene Set Enrichment Analysis

The ARG list was downloaded from the GeneCards database, and then DEARGs were screened from DEGs of GSE13355 and GSE30999. The results showed that 482 and 32 DEARGs existed in GSE13355 (Fig. 3a) and GSE30999 (Fig. 3b) respectively. GO (Table 2) and KEGG (Table 3) functional enrichment analyses were performed on DEARGs in the two datasets respectively. The results of GO showed that DEARGs in GSE13355 were mainly related to leukocyte chemotaxis, cell chemotaxis, leukocyte chemotaxis, and signaling receptor activator activity (Fig. 4a). Meanwhile, the results of KEGG demonstrated that DEARGs in GSE13355 were mainly enriched in viral protein interaction with cytokine and cytokine receptor, cytokine − cytokine receptor interaction, and chemokine signaling pathway (Fig. 4b). Similarly, the GO enrichment of GSE30999 showed that DEARGs was generally enriched in cell chemotaxis, receptor ligand activity, and signaling receptor activator activity (Fig. 4c). The KEGG enrichment of GSE30999 demonstrated that DEARGs were mainly enriched in viral protein interaction with cytokine and cytokine receptor, rheumatoid arthritis, and IL − 17 signaling pathway (Figs. 4d and 5).

**Table 2:** GO enrichment summary

GEO accession	ONTOLOGY	ID	Description	p.adjust
GSE13355	BP	GO:0,071,621	granulocyte chemotaxis	6.96E-14
	BP	GO:0,030,595	leukocyte chemotaxis	1.07E-13
	BP	GO:0,030,593	neutrophil chemotaxis	1.08E-13
	BP	GO:0,097,530	granulocyte migration	1.08E-13
	BP	GO:1,990,266	neutrophil migration	4.03E-13
	CC	GO:0,034,774	secretory granule lumen	0.000156318
	CC	GO:0,060,205	cytoplasmic vesicle lumen	0.000156318
	CC	GO:0,031,983	vesicle lumen	0.000156318
	CC	GO:0,035,580	specific granule lumen	0.000744119
	CC	GO:1,904,724	tertiary granule lumen	0.010042724
	MF	GO:0,008,009	chemokine activity	3.80E-10
	MF	GO:0,042,379	chemokine receptor binding	2.32E-09
	MF	GO:0,005,125	cytokine activity	4.05E-09
	MF	GO:0,048,018	receptor ligand activity	8.87E-08
	MF	GO:0,030,546	signaling receptor activator activity	8.87E-08
GSE30999	BP	GO:0,061,844	antimicrobial humoral immune response mediated by antimicrobial peptide	0.000144534
	BP	GO:0,030,593	neutrophil chemotaxis	0.000391472
	BP	GO:1,990,266	neutrophil migration	0.000391472
	BP	GO:0,019,730	antimicrobial humoral response	0.000391472
	BP	GO:0,071,621	granulocyte chemotaxis	0.000391472
	MF	GO:0,008,009	chemokine activity	0.000206153
	MF	GO:0,042,379	chemokine receptor binding	0.000343157
	MF	GO:0,005,125	cytokine activity	0.001695148
	MF	GO:0,045,236	CXCR chemokine receptor binding	0.004049588
	MF	GO:0,048,018	receptor ligand activity	0.004049588

**Table 3:** KEGG enrichment summary

GEO accession	ID	Description	p.adjust	qvalue	Count
GSE13355	hsa04061	Viral protein interaction with cytokine and cytokine receptor	1.13E-09	9.21E-10	10
	hsa04060	Cytokine-cytokine receptor interaction	1.88E-06	1.53E-06	11
	hsa04062	Chemokine signaling pathway	3.62E-06	2.94E-06	9
	hsa04657	IL-17 signaling pathway	3.62E-06	2.94E-06	7
	hsa05164	Influenza A	0.001870867	0.001518523	6
	hsa04622	RIG-I-like receptor signaling pathway	0.003759724	0.003051647	4
	hsa05160	Hepatitis C	0.008015124	0.006505618	5
	hsa05146	Amoebiasis	0.011253432	0.009134047	4
	hsa04620	Toll-like receptor signaling pathway	0.011253432	0.009134047	4
	hsa05171	Coronavirus disease—COVID-19	0.029315198	0.0237942	5
	hsa05120	Epithelial cell signaling in Helicobacter pylori infection	0.029315198	0.0237942	3
GSE30999	hsa04061	Viral protein interaction with cytokine and cytokine receptor	0.000671079	0.000591225	5
	hsa05323	Rheumatoid arthritis	0.003891546	0.003428479	4
	hsa04657	IL-17 signaling pathway	0.003891546	0.003428479	4
	hsa04060	Cytokine-cytokine receptor interaction	0.027838544	0.024525948	5
	hsa04062	Chemokine signaling pathway	0.034931897	0.030775241	4
	hsa05146	Amoebiasis	0.039637888	0.034921252	3
	hsa04064	NF-kappa B signaling pathway	0.039637888	0.034921252	3

### Gene set enrichment analysis (GSEA)

GSEA was conducted for the two datasets. “c2.cp.kegg.v7.0.symbols.gmt” was selected as the reference gene set and FDR was set to < 0.25, with *p* < 0.05 indicating the significant enrichment pathway (Table 4). The GSEA results showed that GSE13355 mainly existed in up-regulated pathways of allograft_rejection, E2F_targets, and IL6_JAK_STAT3_signaling (Fig. 5a) and was also significantly enriched in down-regulated pathways of androgen_response, bile_acid_metabolism, epithelial_mesenchymal_transition (Fig. 5b). In the same way, the GSEA results of GSE30999 indicated a high similarity with the results of GSE13355 (Fig. 5c and d).

**Table 4:** GSEA enrichment summary

GEO accession	Description	enrichmentScore	p.adjust	qvalues
GSE13355	HALLMARK_UV_RESPONSE_DN	0.68935443	2.32E-14	1.07E-14
	HALLMARK_MYOGENESIS	0.550084315	4.60E-07	2.13E-07
	HALLMARK_EPITHELIAL_MESENCHYMAL_TRANSITION	0.525871045	5.78E-06	2.68E-06
	HALLMARK_ANDROGEN_RESPONSE	0.551362878	0.000681724	0.000315746
	HALLMARK_BILE_ACID_METABOLISM	0.524178209	0.000957271	0.000443368
	HALLMARK_ESTROGEN_RESPONSE_EARLY	0.445622304	0.002960129	0.001371007
	HALLMARK_ADIPOGENESIS	0.4346647	0.004371523	0.002024705
	HALLMARK_APICAL_JUNCTION	0.381580348	0.085395013	0.039551374
	HALLMARK_HYPOXIA	0.375876996	0.091103722	0.042195408
GSE30999	HALLMARK_MYOGENESIS	0.587947	6.57E-07	4.29E-07
	HALLMARK_UV_RESPONSE_DN	0.469064	0.018812	0.012277172
	HALLMARK_ANDROGEN_RESPONSE	0.435298	0.142857	0.093233083
	HALLMARK_BILE_ACID_METABOLISM	0.414859	0.161175	0.105187754
	HALLMARK_KRAS_SIGNALING_DN	0.382145	0.131416	0.085766364
	HALLMARK_ESTROGEN_RESPONSE_EARLY	0.374304	0.181319	0.118334297
	HALLMARK_APOPTOSIS	-0.43498	0.181319	0.118334297
	HALLMARK_P53_PATHWAY	-0.43389	0.148644	0.097009525
	HALLMARK_KRAS_SIGNALING_UP	-0.44073	0.131416	0.085766364
	HALLMARK_GLYCOLYSIS	-0.4613	0.064001	0.041769207

### Construction of protein–protein interaction (PPI) network

**Fig. 6:**
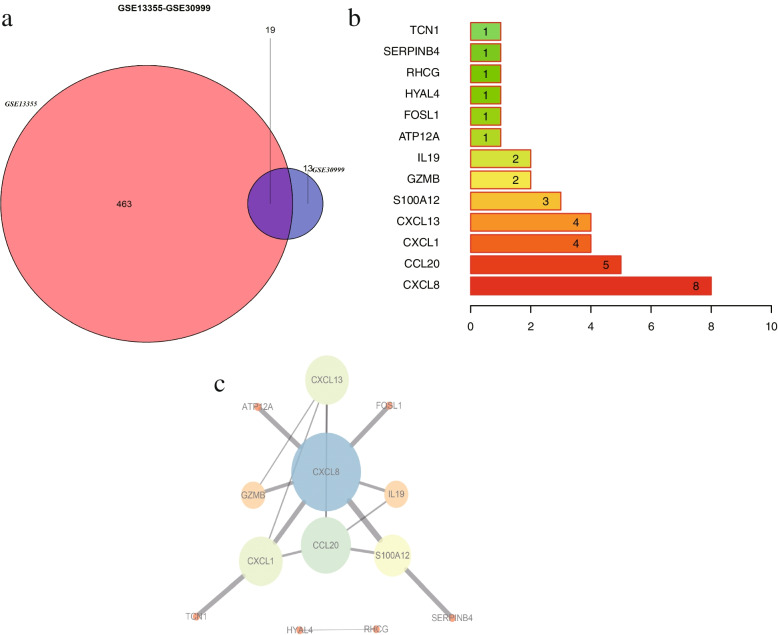
PPI network analysis of common DEARGs. **a** 19 common DEARGs in GSE13355 and GSE30999. **b** Visualizing the number of interaction relationships in each DEARG. **c** Visualizing the different nodes in Cytoscape, in which the larger points represent the greater degrees of nodes and the thicker lines indicate the larger edges. PPI, Protein–Protein Interaction; DEARGs, Differentially Expressed Apoptosis Related Genes

The Venn diagram in Fig. 6a showed that GSE13355 and GSE30999 presented 19 common DEARGs. STRING database was then utilized to conduct PPI analysis on the 19 DEARGs. Finally, 13 DEARGs demonstrated the following PPI relationships: CXCL8, CCL20, CXCL1, CXCL13, S100A12, GZMB, IL19, ATP12A, FOSL1, HYAL4, RHCG, SERPINB4, and TCN1 (Fig. 6b, Table 5, and supplementary data1). The number of interaction was visualized in each DEARG in Fig. 6b. Cytoscape was applied to visualize their network in Fig. 6c.

**Table 5:** The information of 13 DEARGs

Gene Symbol	Count	Change
CXCL8	8	Up
CCL20	5	Up
CXCL1	4	Up
CXCL13	4	Up
S100A12	3	Up
GZMB	2	Up
IL19	2	Up
ATP12A	1	Up
FOSL1	1	Up
HYAL4	1	Up
RHCG	1	Up
SERPINB4	1	Up
TCN1	1	Up

### TF–target, miRNA–mRNA network analysis and drug sensitivity prediction

**Fig. 7:**
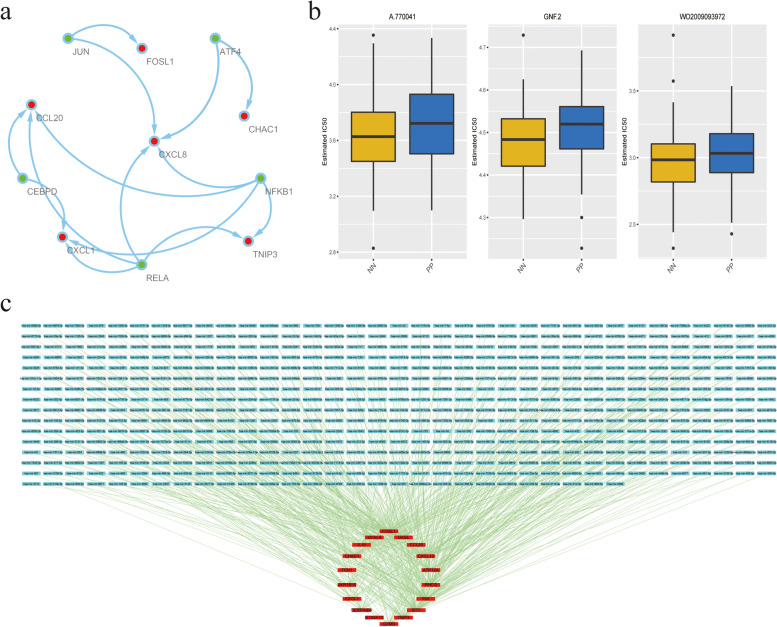
Relevant network construction and drug sensitivity prediction of DEARGs. **a** The TF–target network construction of DEARGs by TRRUST database (Green nodes are predicted TFs, red nodes are DEARGs that can be used as target genes). **b** Visualization of three classes of drugs with *p* < 0.05 (A.770041, GNF.2 and WO2009093972). **c** The miRNA–mRNA regulatory network construction of DEARGs by TargetScan database (Red nodes indicate DEARGs, blue nodes represent associated miRNAs, and green lines stand for the regulatory relationships between DEARGs and miRNAs). DEARGs, Differentially Expressed Apoptosis Related Genes

JUN, ATF4, CEBPD, NFKB1 and RELA were obtained as TFs, with corresponding targets of FOSL1, CHAC1, CCL20, CXCL8, CXCL1, and TNIP3 through the TRRUST database for TF prediction of 19 DEARGs. Their regulatory relationships were visualized as a regulatory network chart (Fig. 7a). The IC50 of 138 drugs was then predicted using the ridge regression model, and the three final classes of drugs with *p* < 0.05 were A.770041, GNF.2, and WO2009093972 (Fig. 7b). Finally, the miRNA of DEARGs was predicted using the TargetScan database. A total of 553 miRNAs were predicted to exhibit regulatory relationships with 18 DEARGs. A network visualization graph was established according to the regulatory relationships (Fig. 7c).

### Relationship of immune infiltration with psoriasis and control samples

**Fig. 8:**
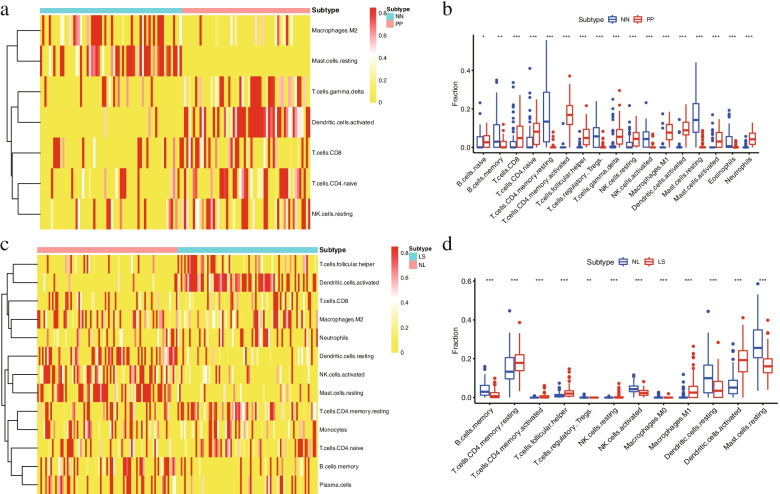
Analysis and visualization of immune infiltration. **a** Heatmap of immune infiltration with high expression in GSE13355. **b** Box line plot of immune infiltration with significant differences in GSE13355. **c** Heatmap of immune infiltration with high expression in GSE30999. **d** Box line plot of immune infiltration with significant differences in GSE30999. (Red represents psoriasis groups, blue represents control groups, *, *p* < 0.05; **, *p* < 0.01; ***, *p* < 0.001). NN, normal skin from controls; PP, involved skin from psoriatic patients; NL, non-lesional skin; LS, psoriasis lesions

Immune infiltration analysis was performed between psoriasis and control samples in GSE13355 and GSE30999 datasets on the basis of CIBERSORT algorithm. An immune infiltration heatmap was illustrated for GSE13355. Only Macrophages.M2, Mast.cells.resting, T.cells.gamma.delta and other cells expressed in most samples were retained in the heatmap. Among them, Macrophages.M2 and Mast.cells.resting presented high levels of infiltration in control groups, while T.cells.gamma.delta, Dendritic.cells.activated, T.cells.CD8, T.cells.CD4.naïve, and NK.cells.resting exhibited high levels of infiltration in psoriasis groups (Fig. 8a). Figure 8b showed the comparison of groups for 22 immune cells with significant differences. The heatmap and comparison of groups for 22 immune cells showed significant differences among Mast.cells.resting, T.cells.gamma.delta, Dendritic.cells.activated, T.cells.CD8, T.cells.CD4.naive, and NK.cells.resting.

The immune infiltration heatmap for GSE30999 showed significant differences among T.cells.follicular.helper, Dendritic.cells.activated, T.cells.CD8, Macrophages.M2, Neutrophils, Dendritic.cells.resting, NK.cells.activated, Mast.cells.resting, T.cells.CD4.memory.resting, Monocytes, T.cells.CD4.naïve. B.cells.memory, and Plasma.cells (Fig. 8c). The combined results of the comparison between groups presented high levels of infiltration of B.cells.memory, T.cells.CD4.memory.resting, NK.cells.activated, and Dendritic.cells.resting in control groups as well as high levels of infiltration of T.cells.follicular.helper, Dendritic.cells.activated, and Mast.cells.resting in psoriasis groups (Fig. 8d).

Overall, Dendritic.cells.activated demonstrated high levels of immune infiltration and Mast.cells.resting exhibited low levels of immune infiltration in psoriasis groups.

### Validation via RT-qPCR

**Fig. 9:**
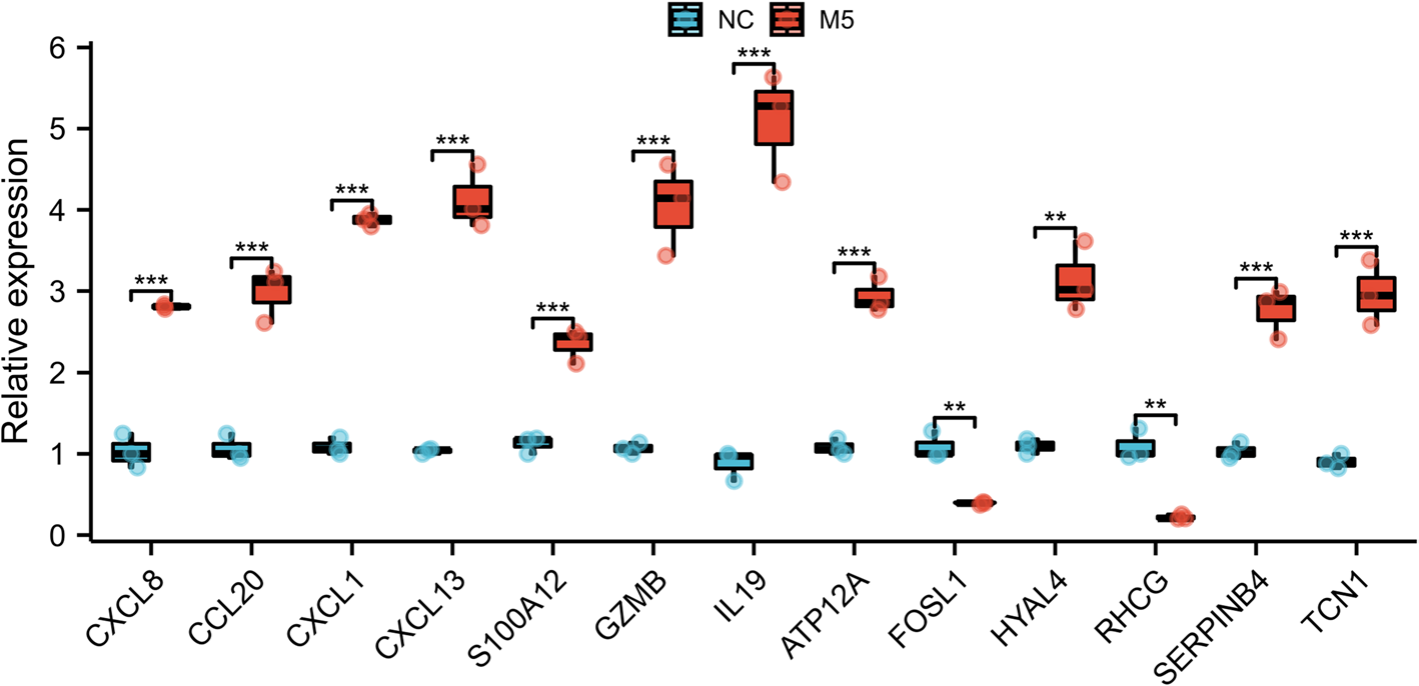
The RT-qPCR results of 13 DEARGs. The transcription levels of CXCL8, CCL20, CXCL1, CXCL13, S100A12, GZMB, IL19, ATP12A, HYAL4, SERPINB4, and TCN1 were significantly up-regulated in M5 groups; however, the transcription levels of FOSL1 and RHCG were down-regulated in M5 groups. (Red represents M5 groups, blue represents normal control groups, *, *p* < 0.05; **, *p* < 0.01; ***, *p* < 0.001). DEARGs, Differentially Expressed Apoptosis Related Genes

Expression levels of 13 key DEARGs were validated using RT-qPCR (*n* = 3). The results of RT-qPCR indicated that the transcription levels of CXCL8, CCL20, CXCL1, CXCL13, S100A12, GZMB, IL19, ATP12A, HYAL4, SERPINB4, and TCN1 were significantly up-regulated and FOSL1 and RHCG were down-regulated in M5 groups (Fig. 9).

## Discussion

An effective cure for psoriasis is still unavailable [4] and its pathogenesis remains poorly defined given that multiple factors come into play [16]. The reductive apoptosis of keratinocytes is a common phenomenon in psoriatic lesions [9, 17]. Many APRs are involved in the pathogenesis of psoriasis [18]. Hence, exploring the molecular mechanism of APRs is necessary to achieve novel therapeutic targets. We identified and further explored the functions of 482 and 32 DEARGs from GSE13355 and GSE30999 respectively, to predict three novel targeted drugs. Meanwhile, characteristics of immune infiltration in psoriasis were comprehensively analyzed.

A total of 514 DEARGs were screened from the two datasets of GSE13355 and GSE30999. GO annotation and KEGG pathway analyses of genes were performed to investigate their further functions. The results of GO annotation showed that DEARGs were typically enriched in cell chemotaxis, receptor ligand activity, and signaling receptor activator activity in both datasets. The results of KEGG pathway indicated that the viral protein interaction with cytokine and cytokine receptor was the pathway with maximum enrichment. These results are different from the findings of previous studies [11, 12, 19]. For example, Choudhary et al. reported that keratinocyte differentiation and positive regulation of cytokine production were the respective biological process and molecular function with maximum enrichment and the cytokines-cytokine receptor was the pathways with maximum enrichment. GSEA analysis for GSE13355 and GSE30999 presented that the high degree of consistency between the results of the two datasets. Thus, the consistency of GSEA results verified the validity of screened DEARGs.

Thirteen key DEARGs with upregulation were derived in the PPI network, including CXCL8, CCL20, CXCL1, CXCL13, S100A12, GZMB, IL19, ATP12A, FOSL1, HYAL4, RHCG, SERPINB4, and TCN1. Eleven key DEARGs, namely, CXCL8, CCL20, CXCL1, CXCL13, S100A12, GZMB, IL19, ATP12A, HYAL4, SERPINB4, and TCN1, were confirmed via RT-qPCR. Among these genes, CXCL8, CCL20, CXCL1, CXCL13, and S100A12 have been extensively investigated [19–21]. Meanwhile, studies on GZMB, IL19 and SERPINB4 are lacking. To the best of our knowledge, ATP12A, HYAL4 and TCN1 in psoriasis remain unverified. Based on the literatures, ATP12A is the nongastric form of H + /K + -ATPase and plays an important role in respiratory diseases [22]. HYAL4 is a member of the hyaluronidases (HYAL) family, which is named as such due to their ability to degrade hyaluronan. HYAL4 is produced by mast cells and plays a particular role in maintaining α-granule homeostasis [23]. Transcobalamin (TCN1) is a vitamin B12-binding protein usually highly expressed in tumor tissues and linked to aggressive tumor behavior and poor prognosis [24].

Nineteen DEARGs were used to construct TF–target and miRNA–mRNA network. The action mechanism of upstream transcription factors and downstream miRNA was easily mined when network relationships of 19 DEARGs were built. This complex network relationships also indicated numerous genes involved in the pathogenesis of psoriasis. Three novel targeted drugs, namely, A.770041, GNF.2, and WO2009093972, were predicted using the ridge regression model. A.770041, a compound with selective inhibitor for Lck, can block T cell activation and IL-2 production [25]. Some studies have shown that A.770041 can function similar to cyclosporin A to prevent acute transplant rejection [25, 26]. GNF-2, an allosteric inhibitor of Bcr-Abl, is a new anticancer drug to treat resistant chronic myelogenous leukemia [27]. At present, the novel drug WO2009093972 still remains unverified. Therefore, A.770041, GNF.2, and WO2009093972 are possible targeted drugs for psoriasis that require further exploration.

Furthermore, characteristics of immune infiltration in psoriasis were analyzed. Various immunocytes involved in psoriasis form a complex network centered on T lymphocytes [15]. Compared with normal control groups, our results showed that high levels of immune infiltration of Dendritic.cells.activated and low levels of immune infiltration of Mast.cells.resting in psoriasis groups. Studies have indicated that dendritic cells are the driver of psoriasis that trigger a series of inflammatory responses [28]. Dendritic cells are the source of cytokines (TNF-α, IFN-γ, etc.) that play a crucial role in the pathogenesis of psoriasis [28, 29].

However, this study presents the following limitations. First, some datasets such as GSE14905, GSE34248, GSE41745, and GSE40033, were not used for analysis due to different sample types or platforms. Second, the sample size in this study was insufficient. Future investigations can attempt to integrate multiple databases to increase the sample size. Third, we lack relevant clinical studies and cannot combine clinical information for analysis. Fourth, although experiments with skin tissue samples were not performed in this study, future explorations should include this, to improve the reliability of the results. Therefore, further studies with additional samples and experiments are required to illustrate the role of key DEARGs and underlying mechanism of psoriasis.

The results of our study may reveal some insights into underlying molecular mechanisms of psoriasis and provide novel targeted drugs.

## Conclusions

This study explored functions of key APRs and analyzed underlying characteristics of immune infiltration of psoriasis through a comprehensive bioinformatics analysis. We identified 13 key DEARGs associated with psoriasis via GEO datasets analysis. Moreover, dendritic cells play an important role in the initiation of psoriasis. Notably, A.770041, GNF.2, and WO2009093972 are possible novel targeted drugs for psoriasis in the future. However, additional experiments are needed to support further our findings.

## Methods

### Data download and preprocessing

Original data of GSE13355 [30] and GSE30999 [31] were downloaded from GEO (https://www.ncbi.nlm.nih.gov/geo/) using the GeoQuery package [32]. All samples in the two datasets were from homo sapiens based on GPL570 ([HG-U133_Plus_2] Affymetrix Human Genome U133 Plus 2.0 Array). The GSE13355 dataset contained 58 lesion skin samples from psoriatic patients and 64 normal skin samples from healthy controls, while the GSE30999 dataset included 85 paired lesion and non-lesion skin samples from psoriatic patients, all of which were included in this study. Original data of GSE13355 and GSE30999 were read using the affy package [33] to obtain their gene expression matrices. Ethical approval is not necessary because this study does not contain any studies with human participants or animals performed by any of the authors.

### Screening and functional analysis of differentially expressed apoptosis-related genes (DEARGs)

The limma package [34] was used to screen differentially expressed genes (DEGs) of GSE13355 and GSE30999. The ggplot2 and pheatmap packages were utilized to illustrate a volcano plot and heatmap of DEGs with a cut-off value setting of adj.*p* value < 0.05 and |log2FC|> 1. The APR list was downloaded from GeneCards database (http://www.genecards.org/) [35], and DEARGs were screened from DEGs. Gene Ontology (GO) and Kyoto Encyclopedia of Genes and Genomes (KEGG) pathway enrichment analyses of DEARGs were performed using the clusterProfiler package [36]. The gene set enrichment analysis (GSEA) of gene expression matrix was also conducted using the clusterProfiler package, and “c2.cp.kegg.v7.0.symbols.gmt” was selected as the reference gene set, while a false discovery rate (FDR) < 0.25 and *p* < 0.05 were considered statistically significantly.

### Protein–protein interaction network construction

The VennDiagram package [37] was applied to illustrate the Venn diagram of DEARGs in GSE13355 and GSE30999. The STRING (https://string-db.org/) database [38] was used to construct the PPI network for common DEARGs in the two datasets, and the NetworkAnalyzer in Cytoscape [39] was utilized to analyze related attributes of nodes in the network.

### TF–target, miRNA–mRNA network analysis, and drug sensitivity prediction

DEARG-related transcription factors (TFs) were predicted using TRRUST database (https://www.grnpedia.org/trrust/) [40], and the TF–target network was visualized via Cytoscape software. A ridge regression model was subsequently used to predict the half-maximal inhibitory concentration (IC50) of 138 drugs through pRRophetic package [41]. Finally, DEARG-associated miRNAs were predicted and a miRNA–mRNA regulatory network was built using TargetScan database(http://www.targetscan.org/vert_71/) [42].

### Analysis of immune infiltration

CIBERSORT algorithm [43] is based on linear support vector regression, deconvolves the transcriptome expression matrix, and thereby estimates the composition and abundance of immunocytes in mixed cells. We uploaded the gene expression matrix to CIBERSORT and filtered out the samples with *p* < 0.05 to obtain the immune infiltration matrix. A heat map was established using the pheatmap package to show the distribution of 22 immunocytes in each sample. Immune infiltration between different subgroups in the two datasets was illustrated using the ggpubr package and visualized at *p* < 0.05.

### Validation with RT-qPCR

Cultured HaCaT cells were treated with 10 ng/ml of M5 (IL-22, TNF-a, IL-17A, IL-1a, and Oncostatin M) (PeproTech) for 48 h. Untreated and treated cells were regarded as normal control (NC) groups and psoriasis cell model (M5) groups respectively. Total RNA was extracted using TRIpure total RNA extraction reagent (#EP013, ELK Biotechnology, China) and reverse transcribed with EntiLink™ 1st Strand Cdna Synthesis Kit (#EQ003, ELK Biotechnology, China). The RNA expression was detected according to the manual of the StepOne™ Real-Time PCR System (Life technologies) using EnTurbo™ SYBR Green PCR SuperMix (#EQ001, ELK Biotechnology, China). Primer sequences were presented in Supplementary Table 1.

### Statistical analysis

R software (version 4.0.0, http://r-project.org/) was used to analyzed the data. T-testing was applied to compare the expression levels of 13 key DEARGs between normal control (NC) groups and psoriasis cell model (M5) groups. The ggplot2 package was utilized to perform the T-testing (*, *p* < 0.05; **, *p* < 0.01; ***, and *p* < 0.001).

## Supplementary Information

**Additional file 1. **

**Additional file 2: Table 1. **The primer sequences of 13 key DEARGs.

## Data Availability

All generated or analysed data during the study are included in this published article.

## Abbreviations

DEARGs: Differentially Expressed Apoptosis Related Genes
GO: Gene Ontology
KEGG: Kyoto Encyclopedia of Genes and Genomes
GSEA: Gene Set Enrichment Analysis
PPI: Protein–Protein Interaction
TF: Transcription Factor
RT-qPCR: Real-time quantitative PCR
APRs: Apoptosis-Related Genes
GEO: Gene Expression Omnibus
DEGs: Differentially Expressed Genes
NN: Normal skin from controls
PP: Involved skin from psoriatic patients
NL: Non-lesional skin
LS: Psoriasis lesions
BP: Biological progress
CC: Cellular component
MF: Molecular Function
