# Supplementary material for: Identification of key apoptosis-related genes and immune infiltration in the pathogenesis of psoriasis
Source: Hereditas. 2022 Jun 22;159:26. doi: 10.1186/s41065-022-00233-0 (PMC9213172; doi:10.1186/s41065-022-00233-0)
Supplement: Supplementary file 2 — Additional file 2: Table 1. The primer sequences of 13 key DEARGs. [file 41065_2022_233_MOESM2_ESM.docx]

**Table 1**

The primer sequences of 13 key DEARGs.

| Gene | Forward primer (5`-3`) | Reverse primer (5`-3`) |
| --- | --- | --- |
| GAPDH | CATCATCCCTGCCTCTACTGG | GTGGGTGTCGCTGTTGAAGTC |
| CXCL10 | AAGTGGCATTCAAGGAGTACCTC | AGCCCTCTTCAAAAACTTCTCC |
| CXCL8 | GACATACTCCAAACCTTTCCACC | CTTCTCCTAAGCGATGCTCAAA |
| CXCL13 | ACATCTCTGCTTCTCATGCTGC | TCCATTCAGCTTGAGGGTCC |
| CCL20 | TTGTCTGTGTGCGCAAATCC | TGGACAAGTCCAGTGAGGCAC |
| S100A12 | ACCAATACTCAGTTCGGAAGGG | GTGGGTGTGGTAATGGGCAG |
| GZMB | GTGCGGTGGCTTCCTGATAC | TCACAGGGATAAACTGCTGGG |
| IL19  ATP12A  FOSL1  HYAL4  RHCG  SERPINB4  TCN1 | GGATCATCAGGAGCCAAACC  TCAGCCAACAGTGAAACAGTGG  AAGCATCAACACCATGAGTGG  TGCACAAGGTGACTAAAGGACC  GACTGGACCGCAAGAACACAG  GAGCTGAAGATCGCCAACAAG  ACTACATCCGCCTAAAACCTCTG | CCAGGCTAGAAAGACGTCGAG  CAATGTCTGCCTTCTTTAGAGCC  CTGATCTGTTCACAAGGCCTTC  AGGAGCCATGAAGTGAGATGTACT  GTAGACCGCATCCTCAAAGCA  CGTTCACAAGAACCAGTGTCGT  GCCAGTATAATCAAGGCAAGCTC |

DEARGs, differentially expressed apoptosis-related genes
